# Consistently processed RNA sequencing data from 50 sources enriched for pediatric data

**DOI:** 10.1038/s41597-025-05376-z

**Published:** 2025-07-02

**Authors:** Holly C. Beale, Katrina Learned, Ellen T. Kephart, A. Geoffrey Lyle, Anouk van den Bout, Molly McCabe, Kathryn Echandia-Monroe, Mansi J. Khare, Elise Y. Huang, Sneha Jariwala, Reyna Antilla, Allison Cheney, Alex G. Lee, Leanne C. Sayles, Stanley G. Leung, Yvonne A. Vasquez, Lauren Sanders, David Haussler, Sofie R. Salama, E. Alejandro Sweet-Cordero, Olena M. Vaske

**Affiliations:** 1 Department of Molecular, Cell and Developmental Biology, University of California Santa Cruz, Santa Cruz, California, USA; 2 Genomics Institute, University of California Santa Cruz, Santa Cruz, California, USA; 3 Division of Pediatric Oncology, University of California San Francisco, San Francisco, California, USA; 4 Division of Radiation Oncology, University of California San Francisco, San Francisco, California, USA; 5 Department of Biomolecular Engineering, University of California Santa Cruz, Santa Cruz, California, USA

**Keywords:** Data integration, Data publication and archiving, RNA sequencing, Paediatric cancer

## Abstract

Larger cohorts improve the power of tumor gene expression analysis, but the signal is muddied if datasets are processed using different methods or have inaccurate metadata. Here we present five compendia containing consistently processed gene expression data derived from 16,446 diverse RNA sequencing datasets. To create the compendia, we obtained access to RNA sequence data from repositories containing public data as well as clinical partners with access to non-published data. We then assessed the quality, quantified gene expression, harmonized clinical metadata, and released the expression values and metadata without access restrictions. These datasets have been used for diverse projects ranging from identifying similarities between tumor types to assessing how well cell lines recapitulate tumors. They have also been used for n-of-1 analysis to identify genes with unusual expression patterns in a single sample and to infer molecular diagnosis. The comparison to new data is enabled by our dockerized, freely available pipeline. The compendia have been cited in at least 20 publications.

## Background & Summary

Gene expression profiling is a powerful tool in cancer research. It is used clinically to distinguish tumor subtypes^1,2^ and to identify potential drug targets^3–7^. For some cancers, it influences clinical decision making, and can predict survival time and likelihood of recurrence^1,8–12^. However, genomic data privacy concerns, computational requirements, technical challenges, and the personnel time involved make assembling large cohorts of tumor data difficult^13^. For rare cancers, such as pediatric cancers, data aggregations from multiple sources are necessary to achieve progress, because the diseases are individually rare and a small cohort of datasets rarely has enough power for sophisticated statistical analysis. We established the Treehouse Childhood Cancer Initiative in 2015 in part to create large compendia of harmonized cancer RNA-sequencing datasets, focused on pediatric cancers, to enable state-of-the-art genomic studies in pediatric oncology.

Our first compendium was compiled in 2016. It included seven pediatric tumor types and 10,368 datasets, where a dataset is data generated from one sample. It was based on our UCSC Genomics Institute colleagues’ uniform processing of RNA-Seq datasets^14^ from NCI’s The Cancer Genome Atlas (TCGA)^15^ and the corresponding NCI pediatric study Therapeutically Applicable Research To Generate Effective Treatments (TARGET)^16–20^.

**Fig. 1:**
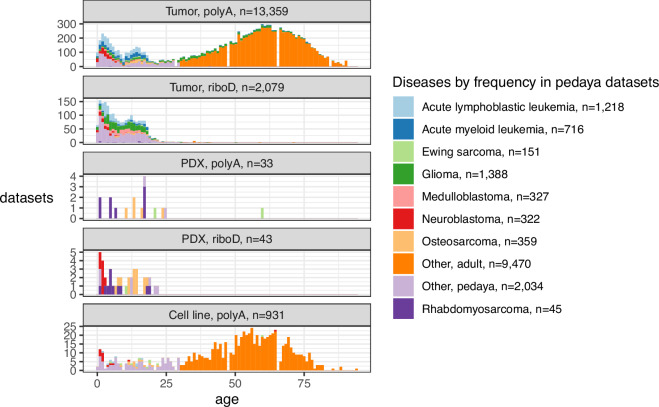
Distribution of ages in compendia. The three most common diseases in pediatric, adolescent and young adult (pedaya, age <= 30) datasets in each compendium are specified by color. Datasets without associated ages (n = 974) are excluded from this plot.

Since then, we have worked to increase the number of pediatric cancer datasets in our compendia. Currently, we have five compendia, including those dedicated to PDX, cell and tumor data, divided by transcript selection method. The compendia now include 16,446 datasets, 5,687 of which are from pediatric, adolescent and young adult individuals (pedaya, Fig. 1). The pediatric datasets span 128 cancer classifications as defined by International Classification of Diseases for Oncology, 3rd Edition, ICD-O-3^21^; 31 classifications are represented by 20 or more datasets. The compendia contain datasets from large sequencing projects such as St. Jude, CBTN/Kids First DRC, and ICGA as well as many smaller single-study projects (Table 1).

**Table 1:** Sources of data.

Source name	n	percent of all datasets
The Cancer Genome Atlas Program (TCGA)^15^	9806	59.6%
St. Jude^34–37^	2142	13.0%
Therapeutically Applicable Research to Generate Effective Treatments (TARGET)^16–20^	1356	8.2%
Cancer Cell Line Encyclopedia (CCLE)^38^	893	5.4%
Children’s Brain Tumor Network/Kids First Data Resource Center^39,40^	377	2.3%
International Cancer Genome Consortium (ICGC)^41–44^	191	1.2%
Clinical collaborators	677	4.1%
Other projects via database of Genotypes and Phenotypes (dbGaP) /Short Read Archive (SRA)^45–64^	801	4.9%
Other projects via European Genome-phenome Archive (EGA)^65–71^	203	1.2%

**Fig. 2:**
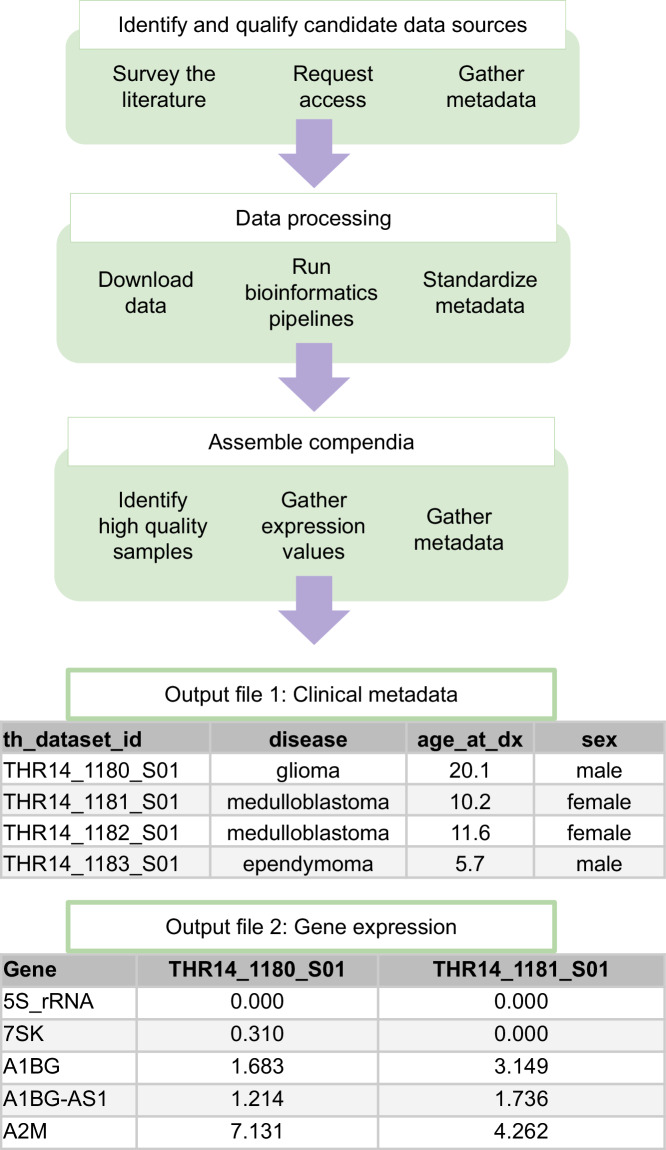
Steps for assembling a gene expression data compendium derived from RNA sequencing.

To achieve this increased representation of pediatric diseases and types of data in the compendia, we identified candidate datasets by surveying scientific literature and data repositories for studies of gene expression in tumors, PDX and cell lines (Fig. 2). To consider data for inclusion in a compendium, we must know (1) the biological source type, such as tumor, PDX, or cell lines; (2) the method for transcript selection; (3) whether it is paired-end, (4) the kind of cancer, and (highly preferably) (5) the age or pediatric status of the person the cancer was found in. We assessed whether the data is comparable with existing compendia, e.g. does it share a transcript enrichment method and biological source type with an existing compendium? All compendia contain only paired-end data. Access to the sequence data for most of the datasets was controlled and required a multi-step application process. In our applications, we requested permission to redistribute the gene expression values we obtain through processing the sequence data. If we were granted access under these terms, we downloaded the metadata provided by the repository and searched literature for additional metadata. We also obtained datasets from collaborators that had not deposited the data into public repositories, and contributed data from 12 samples we sequenced ourselves. In all of our access requests, we asked to make de-identified aggregate gene expression measurements available to the public on our website.

Unfortunately, datasets were excluded at each step of the process due to incomplete, inaccurate or missing metadata, underlining the need for simpler access methods and careful consideration of usability before releasing data^13^. For example, crucial information like transcript enrichment method and disease were not always available in SRA. For one SRA study, we reviewed 4 publications to obtain transcript enrichment method and disease, and yet disease remained undetermined in 7 (nearly 10%) of the datasets. In other SRA records, age and sex were not available on SRA, in the publication, or by communication with the authors. In direct communications, we have received incorrect information about the transcript enrichment method, which was then corrected when we observed high abundance of non-polyadenylated histone transcripts in a nominally polyA-selected dataset. Even when accurate, well-structured metadata is available in a repository, access can be challenging. An EGA dataset had associated articles referring to both RNA-Seq and whole genome sequencing (WGS) data. Only after applying for access to the controlled access dataset were we able to determine that the data was WGS.

If the datasets were sufficiently annotated and appropriate for our compendia, we next added the metadata to our secure REDCap database. If we had permission, we downloaded the FASTQ or BAM sequence data files and processed the sequence data with our dockerized pipeline, generating quality metrics and gene expression quantification values. When we did not have permission to download the raw sequence data, we had two options to generate fully comparable output data. Three institutional partners used option A: deploying our dockerized pipeline to process the sequence data in their own ecosystem and sharing the output with us for incorporation into our compendia. With one institutional partner we used option B: implementing our dockerized pipeline on the institution’s cloud-based platform, processing the data ourselves, and downloading the output. Both of these avenues eliminate the need for raw data transfer and represent important options for harmonizing genomic data, given regulatory and technical restrictions.

Compendia were assembled from comparable gene-level expression data and paired with clinical metadata such as disease type, sex, and age. Data were considered comparable if the same input RNA selection method was used and biological sample types were deemed similar by our analysts. Sequence data generated from poly-A selected RNA and ribo-depleted RNA are in separate compendia, as are data from different sample types such as tumors, *in vitro* cell cultures, and patient-derived xenograft (PDX) models. The compendia files were then shared with the public via GEO and other sources (see Usage Notes section).

## Methods

### Data identification

Adult, pediatric, adolescent, and young adult RNA-Seq data and clinical metadata are identified in repositories including Database of Genotypes and Phenotypes (dbGAP/SRA), European Genome-phenome Archive (EGA), Sequence Read Archive (SRA), St. Jude Cloud, and Kids First Data Resource Center, as well as through direct collaborations. Metadata are analyzed to determine whether the data are comparable, and permissions for access are sought.

### Metadata processing

The diseases are harmonized, and age and source are compiled and recorded in a REDCap database^22^. ICD-O-3 harmonized disease values are determined by Treehouse researchers when they are not provided.

### RNA extraction, library preparation and sequencing

Twelve QC pass datasets were sequenced internally. TH46_2416_S01 data was generated from a flash frozen tissue sample as previously described^4^; the RNA had a RIN score of 5.7. For the 5 datasets with IDs TH44_4659_S02-TH44_4659_S06, RNA was extracted from Formalin-Fixed Paraffin-Embedded (FFPE) tumor tissue samples using the truXTRAC FFPE total NA (tNA) Ultra Kit - Column (Covaris) and the ME220 Ultrasonicator System for Adaptive Focused Acoustics (Covaris). For the 6 datasets with IDs TH46_2416_S02, TH46_5187_S01, TH46_5187_S02, TH46_5188_S01, TH46_5188_S02, and TH46_5189_S01, RNA was obtained from flash frozen tissue homogenized in DNA/RNA Shield using ZR BashingBead Lysis Tubes (Zymo) and extracted using Quick DNA/RNA miniprep Kit (Zymo). For all 11, RNA quantity was measured using the Qubit™ High Sensitivity RNA Assay (Thermo Fisher Scientific) and the RNA quality was measured using the Tapestation High Sentisitivity RNA assay (Agilent). The RNA libraries were constructed using the Stranded Total RNA Prep, Ligation with Ribo-Zero Plus Kit (Illumina). Ribosomal RNA was enzymatically depleted from 50–100 ng of total input RNA with a DV200 score of 55% or higher. Reverse transcription was used to convert RNA into cDNA followed by A-tailing, adapter ligation and amplification. The RNA libraries were sequenced using the NextSeq. 1000 System (Illumina). The 200 cycle kit (Illumina) was used to generate 100 bp paired-end sequencing reads and 70–95 million reads per sample. PhiX Control v3 was used as a sequencing run quality control and spiked in at 5%.

### Sequence data processing

Gene expression in each sample is uniformly quantified using the dockerized TOIL RNA-Seq pipeline versions from 3.2 to 3.4.1^14^; all of these versions produce bitwise identical RSEM gene expression outputs. The pipeline uses RSEM Version 1.2.25^23^ for quantification after aligning reads with STAR v 2.3.2a^24^ using indices generated from the human reference genome GRCh38 and the human gene models GENCODE 23 as described at https://github.com/UCSC-Treehouse/pipelines. Stranded and non-strand-specific data is processed identically. Quality is assessed with the MEND pipeline https://github.com/UCSC-Treehouse/mend_qc^25^.

### Compendia assembly

The metadata database is surveyed to identify datasets relevant to each compendium. The RNA-Seq data must contain at least 10 million mapped exonic, non-duplicate (MEND) reads. Samples are de-duplicated as follows: only one of any set of technical replicates are included (or technical replicates are combined); nominally unrelated samples that have extremely high correlation are investigated as potential duplicates; and samples from the same tumor that might have different biology (e.g. different loci from the same tumor) are retained.

Gene expression outputs from RSEM and tab delimited metadata are processed by the compendium generation script, with arguments to select the desired unit of expression. We also generate a file containing the assignment of colors to diseases to maintain consistency in visualizations across releases and visualization tools.

Compendia names reflect sample type (tumor, cell lines, PDX) and input RNA type (i.e. poly-A RNA, ribo-depleted RNA). The version of the compendium is named with the year and month of publication (for compendia generated in 2021 or later) or sequentially (for compendia generated before 2021).

### Compendia distribution

The compendia are published on the NCBI Gene Expression Omnibus (GEO; https://www.ncbi.nlm.nih.gov/gds) and as described in the Usage Notes section.

## Data Records

The compendia are available in the NIH’s gene expression omnibus (GEO) repository with the accession numbers in Table 2. Each compendium consists of a metadata table, and two gene expression tables. Gene expression tables contain one row per gene and one column per RNA-Seq result (one dataset). The expression values are in units of log2(TPM + 1) and expected counts. The corresponding metadata table contains one row per dataset identifier and one column for each type of metadata. In Tables 3, 4 below, we summarize the values found in the metadata tables for each compendia.

**Table 2:** Features of each compendium.

Sample type	Library preparation method	GEO accession	Version	Citation	Datasets
Tumor	polyA selection	GSE294351	25.01	^72^	13,359
Tumor	ribosomal RNA depletion	GSE294353	25.01	^73^	2,079
Cell line	polyA selection	GSE294350	21.06	^74^	932
PDX	polyA selection	GSE294349	22.03	^75^	33
PDX	ribosomal RNA depletion	GSE294352	22.03	^76^	43

**Table 3:** Metadata for tumor and PDX compendia.

Metadata field	Column name	Values
Treehouse dataset identifier	th_dataset_id	[study_id]_[donor number]_S[dataset within donor], e.g. “THR37_1294_S01”
Treehouse harmonized disease	disease	one of 192 values, e.g. “acute lymphoblastic leukemia”, “Ewing sarcoma”
ICD-O disease code	icd_disease	one of 139 values, e.g. “9801/3: Acute leukemia, NOS”, “9364/3: Ewing sarcoma” or “unavailable”
Age at diagnosis, in years	age_at_dx	0–90, e.g 1.9 or “unknown”
Organism	organism	“Homo sapiens”
Is the patient pediatric, adolescent or young adult?	pedaya	“yes”, “no”, or “unknown”
Sex	sex	“female”, “male”, or “unknown”
Treehouse code for the source of a group of datasets, used in the Treehouse dataset identifier	study_id	typically represents a research group or publication, usually starting with TH (for clinical partners) or THR, e.g. “TH03”, “THR37”; exceptions include “TCGA” and “TARGET”
Repository ID for the source of a group of datasets	study_accession	Study ID from SRA, EGA, dbGaP or St Jude. e.g. “SRP092501”, “EGAD00001001098”, or “unavailable”
Name of the repository or host of the study	source_name	e.g. “St. Jude Cloud”; or “unavailable”
Identifier used by the repository or publication to refer to the individual who donated the tissue	study_donor_id	e.g. “TARGET-30-PASSRS”, “BZ11-Tumor”, “RMS1”; or “unavailable”
Identifier used by the repository or publication to refer to RNA-Seq data generated from one sample	study_dataset_id	e.g. “EGAN00001179688”, “SRR4306220”; or “unavailable”

**Table 4:** Metadata for cell line compendia.

Metadata field	Column name	Example values
Treehouse dataset identifier	th_dataset_id	“TH03_0028_S03”, “G20461”, “TARGET-52-NAAELV-50A-01R”
Disease that caused the tumor from which the cell line is derived	disease	one of 171 values, e.g. “medulloblastoma”, “embryonal rhabdomyosarcoma”
Age in years	age	0.25–94; NA
Sex	sex	“female”, “male”, or “unknown”
Treehouse code for the source of a group of datasets	study_id	“CCLE”, “TARGET”, “TH27”, “THR13”
DepMap ID (previously Model ID)	depmap_model_id	“ACH-000058”, “ACH-000372”, “NA”
Identifier used by the source to refer to the cell line	study_dataset_id	“HSC-3”, “KMM-1”
CCLE ID	ccle_id	“HSC3_UPPER_AERODIGESTIVE_TRACT”, “HS895T_SKIN”, “NA”
Unique identifier associated with the RNA-Seq dataset	rnaseq_uuid	“f4f9be10-fb95-40db-868f-d7714c9b4203”, “NA”
RNA-Seq file name	rnaseq_file_name	“G20461.HSC-3.2.bam”, “NA”
American Type Culture Collection (ATCC) cell line identifier	atcc_identifier	“HTB-24”, “CRL-7598”, “NA”
Anatomical collection site	tissue	“upper_aerodigestive_tract”, “pancreas”, “NA”
Corresponding TCGA cohort	corresponding_tcga_cohort	“DLBC”, “ESCA”, “NA”
Histology	histology	“lymphoid_neoplasm”, “anaplastic astrocytoma”, “NA”
Organism	organism	“Homo sapiens”

### Tumor and PDX compendia

For tumor and PDX compendia, whether containing polyA-selected data or ribo-depleted datasets, every record has an associated disease annotation (Table 3). Most also have age at diagnosis, sex, the study the data originate from and additional dataset identifiers from the source. All values except age are encoded as strings.

### Cell line compendia

Metadata for cell lines differ from tumors (Table 4). Because most (896/932) of our current cell line compendium comes from CCLE, we’ve included the information needed to match CCLE metadata to sequencing data. They include the parent cell line, CCLE identifiers, and corresponding TCGA cohort where relevant. All values except age are encoded as strings.

## Technical Validation

Each dataset added to our compendia is subject to rigorous technical validation. We define the technical quality of the dataset on three levels: (1) the quality of the sample preparation and sequencing; (2) accurate metadata; and (3) the impact of batch effects.

First, we address sample and sequencing quality issues. Sample volume, tissue storage and library preparation can lead to low quality RNA-Seq data, and we rarely have access to the quality metrics that would inform us about the details of each step^26,27^. Instead we infer the quality from the sequencing data. Specifically, we count the number of MEND reads, and use a threshold (10 million MEND reads) that infers a reasonable degree of quality at each step^25^. Here and subsequently, all references to read counts refer to the number of pairs of reads. Our MEND count threshold is based on the observation that (1) the median number of MEND reads present in a survey of more than 2000 RNA-Seq datasets was 50% of total reads and (2) the average of two major recommendations for sequencing depth (10 million reads from ’t Hoen *et al*.^28^ and 30 million reads from ENCODE Project Consortium^29^) is 20 million reads. Since there are 10 million MEND reads in a typical RNA-Seq dataset of 20 million reads, we set a threshold of at least 10 million MEND reads to accept a dataset into a compendium. We do not consider 10 million MEND reads a recommended depth but a minimum depth at which the dataset is informative.

Secondly, we address metadata accuracy issues by a combination of careful up-front curation and diligent follow-up of anomalies^13^. When publications and repositories report different metadata for the same samples, our curation team reaches out to originators to resolve the contradiction. Consistently inaccurate metadata, on the other hand, can be difficult to detect. The most common inaccuracy is incorrect reporting of the library preparation method. We review each compendium using the TumorMap visualization tool, which arranges datasets according to similarity of expression values^30^. On TumorMap, we have seen datasets from one study remain separate from datasets we would expect to be similar based on diagnosis. We reach out to the data originators, explain our observations, and ask them to reconfirm their metadata. In this way, some datasets were ultimately determined to have been generated via a ribosomal RNA depletion library preparation in spite of the initial report that they were generated via polyA selection.

Metadata errors have also been detected during clinical analysis using our Comparative Analysis of RNA Expression (CARE) pipeline^7^. For example, in one dataset reported to be generated via polyA selection, many non-polyadenylated genes were identified as exceptionally highly expressed relative to the compendium of polyA-selected tumor datasets. However, data had actually been generated via a ribosomal RNA depletion library preparation, and thus the apparent high relative expression was an artifact of the inclusion of non-polyadenylated transcripts. Datasets determined to be in the wrong compendium are removed.

Anomalies can be due to inaccurate metadata, or they can indicate complex biological phenomena. CARE reported that a sample from a teratoma patient was most similar to glioma or glioblastoma. We discussed the finding with the clinician, and a histological review identified glial features. The patient was subsequently diagnosed with gliomatosis peritonei, which has a more favorable prognosis than teratomas without mature glial tissue^6^.

**Fig. 3:**
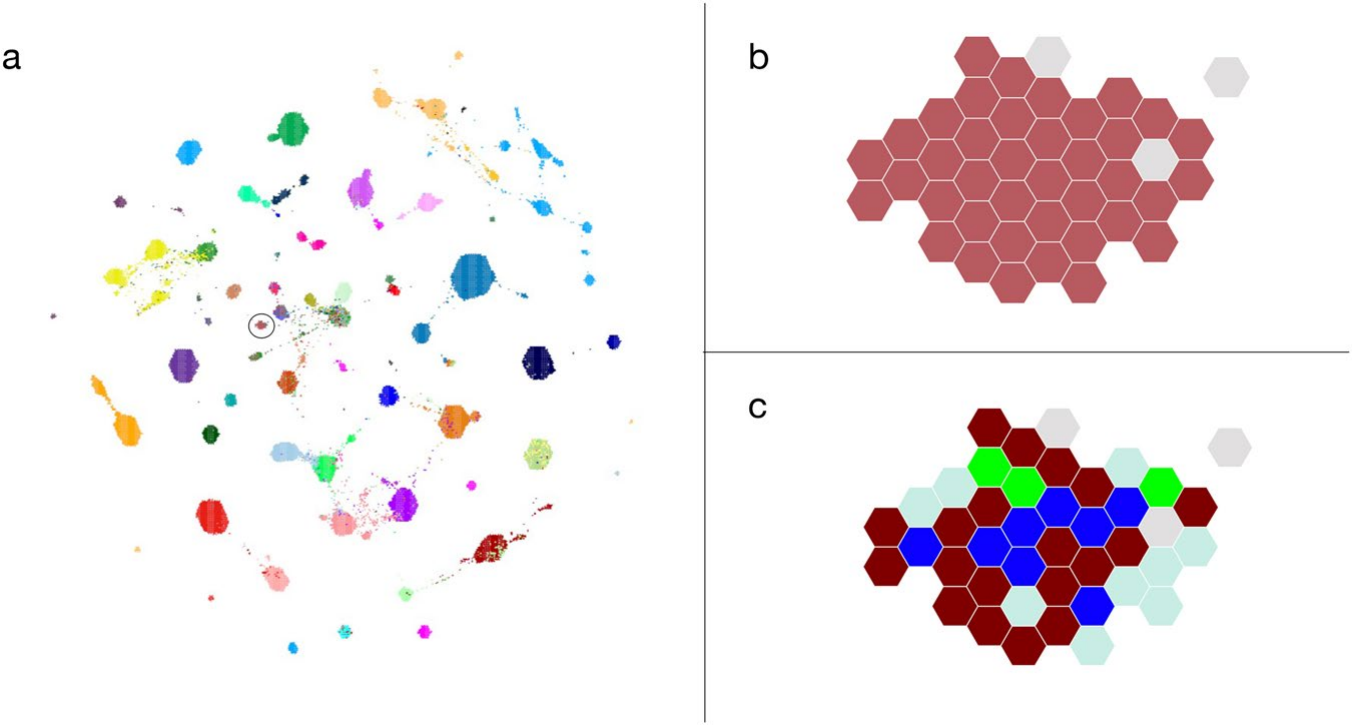
TumorMap visualization of datasets in the PolyA Tumor compendium version 11. (**a**) All 12,747 datasets; each point represents one dataset. Position is based on similarity of gene expression. Colors indicate the diagnosis of the donor. The circled group are mostly synovial sarcomas. (**b**) Synovial sarcoma and related datasets (red are synovial sarcoma; gray are other diseases). (**c**) Study sources of the synovial sarcoma datasets: SRP126664 (brown), phs000178 (light blue), phs000673.v2.p1 (green), data from two unrelated collaborators that was unpublished at the time of the compendium release (blue).

The third aspect of our technical validation concerns batch effects. Because our data come from different sources (often corresponding to institutions), we assume some batch effects are present. Unfortunately, using a batch effect removal tool like ComBat removes biological signals as well as batch effects^31^. Instead, when adding datasets, we visualize their correlations to datasets from other sources using TumorMap. For example, we consider the likelihood of batch effects low if the new datasets are dispersed among those from other sources. Figure 3 shows a TumorMap grouping of more than 30 synovial sarcoma datasets that come from four sources. The interspersed placement indicates that the similarity of the datasets is greater than batch effects that might divide them. If, on the other hand, data for a given cancer from one source are consistently placed separately from data from the same cancer from other sources, we revisit the metadata as described above to confirm the datasets are assigned to the correct compendium. If the metadata appears correct, we leave the data in the compendium because the distinct placement may be due to biological phenomena. We encourage users to review correlations across institutions to assess the possible role of batch effects in their analyses.

## Usage Notes

Internally, we use our compendia to identify potential druggable targets for individual pediatric cancer patients. Our Comparative Analysis of RNA Expression (CARE) identifies transcripts in a patient’s tumor that have exceptionally high expression relative to all tumors in a particular compendium (pan-cancer analysis) or relative to similar tumors (pan-disease analysis)^7^. We have analyzed RNA-Seq tumor data from 164 patients and identified clinically relevant genomic information for 129^3,4,6,7,25^. For at least 8 of the patients, this information was applied, and six of them benefited^3,4,6,7^.

We and others have used the compendia for a variety of purposes (Table 5). In 2024, our compendia averaged 80 downloads per month.

**Table 5:** Research employing Treehouse compendia.

Use of compendia	Publication(s)
Identify potential druggable targets for individual pediatric cancer patients	^3,4,7^
Provide diagnostic classification for individuals	^6,77,78^
Predict therapies for cohorts of patients	^79–82^
Identify targets for drug development and repurposing	^83,84^
Identify mechanisms of drug resistance in tumors	^85^
Characterize the effects of a novel fusion found in an individual with a rare cancer type	^86^
Identify similarities between tumor types	^87^
Assess how well cell lines represent tumors	^88,89^
Improve how well cell lines can represent tumors	^90^
Assess how well normal tissue can be used as a comparison to tumor tissue	^91^

Our website (https://treehousegenomics.soe.ucsc.edu/public-data) hosts an overview of all compendia, with links to sources for (1) downloading the data GEO (https://www.ncbi.nlm.nih.gov/gds) and Xena (https://xena.treehouse.gi.ucsc.edu)^32^ and (2) visualizing data without downloading it: the Tumor Map (https://Tumormap.ucsc.edu/?p=Treehouse/TumorCompendium_v11_PolyA), Xena (https://xena.treehouse.gi.ucsc.edu)^30^ and the UCSC cell browser (https://cells.ucsc.edu/?ds=treehouse)^33^.

As large aggregations of consistently processed data, gene expression compendia are resource-intensive to assemble. They require ongoing attention to resolve inconsistencies, perform rigorous quality control and incorporate newly available data. However, these efforts have proven worthwhile, yielding contributions to clinical and translational cancer research. Through assembling these data and making them easily accessible, we aim to reduce barriers to entry into the field of pediatric oncology, increase the ability of researchers to make accurate and meaningful contributions, and ultimately advance the wellbeing of children with cancer.

## Data Availability

Compendia can be generated from a collection of single-dataset expression files using the build_compendium_matrix.py script available on GitHub at https://github.com/UCSC-Treehouse/compendium-expression-matrix and archived at 10.5281/zenodo.15213775.
